# In memory of Dr. Takashi Sugimura, 1926–2020

**DOI:** 10.1186/s41021-020-00169-9

**Published:** 2020-12-01

**Authors:** Masami Yamada

**Affiliations:** National Defense Academy of Japan, Yokosuka, Japan


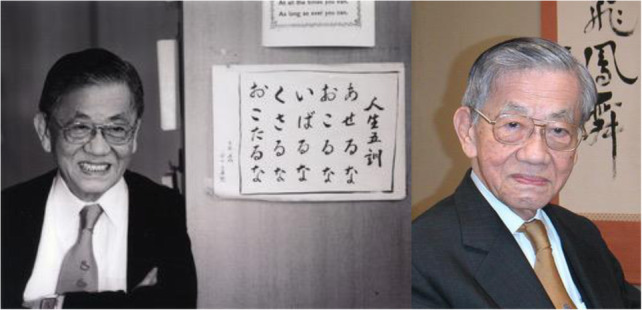


Dr. Takashi Sugimura, an honorary member of the Japanese Environmental Mutagen Society (JEMS), passed away on September 6, 2020, at the age of 94. Dr. Sugimura was a leading international figure in cancer medicine for many years, and was a pioneer in molecular biology. He graduated from the University of Tokyo, Faculty of Medicine, in 1949, received his Ph.D. in 1957, and went abroad to study at the National Cancer Institute in the United States. He then joined the National Cancer Center Research Institute in Tokyo in 1962 and was appointed Director in 1974. He later concurrently served as a Professor of Molecular Oncology at the Institute of Medical Science in the University of Tokyo from 1970 to 1988. He was appointed to be President of the National Cancer Center in 1984 and was instrumental in promoting the government’s Comprehensive 10-Year Strategy for Cancer, which was launched that year, and was appointed Honorary President of the National Cancer Center in 1992. He also served as President of Toho University from 1994 to 2000, and was named President Emeritus in 2000. In 2003, he was diagnosed with stomach cancer and had his entire stomach removed. In an effort to help other patients and their families, he wrote a book about the testing and treatment process and his life after surgery, and continued to work on the development of cancer treatments from the patient’s perspective.

Dr. Sugimura’s research clarified the relationship between mutagenicity and carcinogenicity and produced major contributions to the scientific study of cancer development mechanisms. He is especially remembered for his work on the now-well-known fact that cancer is a disease caused by genetic mutations. Together with Dr. Minako Nagao (who later became Chief of the Carcinogenesis Division) and other researchers, he showed that common cooking practices can produce mutagens exhibiting the same structures as those of heterocyclic amines in food. He then isolated and identified a large number of these heterocyclic amines, and showed that they are taken into the body via food consumption and induce mutations in genes, resulting in the development of tumors. He also analyzed multi-step carcinogenesis at the molecular level and studied its relevance to potential avenues for the prevention of cancer. This research has significantly impacted the identification of new mechanisms of chemical carcinogenesis and tumor promotion.

Dr. Sugimura was the second President of JEMS for 4 years from 1978 and was the chairman of 5th meeting of JEMS hosted in 1976 in Tokyo. In 1981, he was a President for Third International Conference of Environmental Mutagens, which was held in Tokyo Japan and hosted by JEMS on behalf of International Association of Environmental Mutagen Societies (IAEMS, now IAEMGS). He also became an honorary member of JEMS in 1987, and was a frequent lecturer at annual JEMS meetings. In addition, in 1994, when the JEMS Award was established, the first prize was awarded for his “Research on the mutagenicity and carcinogenicity of heterocyclic amines.”

The International Symposium of the Princess Takamatsu Cancer Research Fund, held in Tokyo every autumn, introduces the in latest cancer research and provides a forum for interaction between foreign and domestic researchers. Dr. Sugimura served as the Chair of the Scientific Advisory Committee for this fund for 25 years, from 1989 to 2014, and made significant contributions to the Fund’s activities, including playing a key role in establishing the AACR Princess Takamatsu Memorial Lecture Prize in 2007. He was also instrumental in the establishment of the successful Joint Conference of the American Association for Cancer Research and the Japanese Cancer Association, which began in 1989, and he co-chaired the first meeting with the former President of the AACR, Dr. Enrico Mihich. This joint conference is held every 3 years in Hawaii, and continues to symbolize the close relationship between cancer science researchers in Japan and the United States.

Dr. Sugimura’s work has been recognized in the United States as well in Japan, appearing five times on the cover of Cancer Research. He has also been the recipient of multiple honors in the United States, with his election as a member of the AACR in 1969, an honorary member in 1980, and the first fellow of the AACR Academy in 2013. His accomplishments are too numerous to list in full here, so I will only list some of the major awards he has received as evidence of his high esteem: the Imperial Prize and Japan Academy Prize in 1976, the EMS Award from the Environmental Mutagen Society (U.S.A.; now the Environmental Mutagenesis and Genomics Society) in 1978, the Order of Culture of the Japanese Government in 1978, the Charles S. Mott Prize from the General Motors Cancer Research Foundation in 1981, the Tomizo Yoshida Prize from the Japanese Cancer Association in 1992, and the Japan Prize for Biotechnology in Medicine in 1997. He also served as the 25th President of the Japan Academy in 2013–16. He was an elected foreign associate of the National Academy of Sciences and the Institute of Medicine (U.S.), and an elected foreign member of the Royal Netherlands Academy of Arts and Sciences and the Royal Swedish Academy of Sciences.

There is an expression in Japanese, “fall of a giant star.” In the field of genotoxicity and cancer research, Dr. Sugimura’s death may be best described using this expression. It is an undeniable fact that Dr. Sugimura was and will continue to be respected for his intellectual contributions to research, his amazing leadership skills, and his supreme passion for research above all.

May he rest in peace.

Editor-in-Chief

Masami Yamada

National Defense Academy of Japan

